# Neutrophil extracellular traps impair intestinal barrier functions in sepsis by regulating TLR9-mediated endoplasmic reticulum stress pathway

**DOI:** 10.1038/s41419-021-03896-1

**Published:** 2021-06-11

**Authors:** Shilong Sun, Zehua Duan, Xinyu Wang, Chengnan Chu, Chao Yang, Fang Chen, Daojuan Wang, Chenyang Wang, Qiurong Li, Weiwei Ding

**Affiliations:** 1 Division of Trauma and Surgical Intensive Care Unit, Research Institute of General Surgery, Affiliated Jinling Hospital, Medical School of Nanjing University, Nanjing, 210002 Jiangsu P. R. China; 2 Key Laboratory of Intestinal Injury, Research Institute of General Surgery, Affiliated Jinling Hospital, Medical School of Nanjing University, Nanjing, 210002 Jiangsu P. R. China; 3 School of Medicine, Southeast University, Nanjing, 210009 P. R. China; 4 State Key Laboratory of Analytical Chemistry for Life Science & Jiangsu Key Laboratory of Molecular Medicine, Medical School of Nanjing University, Nanjing, 210093 Jiangsu P. R. China; 5 The First School of Clinical Medicine, Southern Medical University, Nanjing, 210002 Jiangsu P. R. China

**Keywords:** Mucosal immunology, Intestinal diseases, Sepsis

## Abstract

Increased neutrophil extracellular traps (NETs) formation has been found to be associated with intestinal inflammation, and it has been reported that NETs may drive the progression of gut dysregulation in sepsis. However, the biological function and regulation of NETs in sepsis-induced intestinal barrier dysfunction are not yet fully understood. First, we found that both circulating biomarkers of NETs and local NETs infiltration in the intestine were significantly increased and had positive correlations with markers of enterocyte injury in abdominal sepsis patients. Moreover, the levels of local citrullinated histone 3 (Cit H3) expression were associated with the levels of BIP expression. To further confirm the role of NETs in sepsis-induced intestinal injury, we compared peptidylarginine deiminase 4 (PAD4)-deficient mice and wild-type (WT) mice in a lethal septic shock model. In WT mice, the Cit H3-DNA complex was markedly increased, and elevated intestinal inflammation and endoplasmic reticulum (ER) stress activation were also found. Furthermore, PAD4 deficiency alleviated intestinal barrier disruption and decreased ER stress activation. Notably, NETs treatment induced intestinal epithelial monolayer barrier disruption and ER stress activation in a dose-dependent manner in vitro, and ER stress inhibition markedly attenuated intestinal apoptosis and tight junction injury. Finally, TLR9 antagonist administration significantly abrogated NETs-induced intestinal epithelial cell death through ER stress inhibition. Our results indicated that NETs could contribute to sepsis-induced intestinal barrier dysfunction by promoting inflammation and apoptosis. Suppression of the TLR9–ER stress signaling pathway can ameliorate NETs-induced intestinal epithelial cell death.

## Introduction

Sepsis is defined as life-threatening organ dysfunction caused by the dysregulation of the host response secondary to infection, which remains a leading cause of high mortality in the intensive care unit [1, 2]. The gastrointestinal tract is the most easily and frequently involved organ in the process of sepsis and the gut has been deemed the motor of sepsis [3, 4]. The breakdown of the gut barrier can result in many bacteria and toxins entering the internal environment, driving lethal sepsis and even multiple organ dysfunction syndrome (MODS) [5]. Hence, a more thorough understanding of the inflammatory mechanism involved in intestinal barrier dysfunction is pivotal for developing more efficient treatments against sepsis and MODS.

Since neutrophil extracellular traps (NETs) were firstly described by Brinkman in 2004, excessive NETs formation has been shown to be involved in the pathophysiology of sepsis [6, 7]. NETs are web-like structures protruding from the membrane of activated neutrophils, comprising decondensed DNA fibers accompanied by intracellular proteins, including histones, myeloperoxidase (MPO), and other antimicrobial proteins [6]. Histone citrullination induced by peptidylarginine deiminase 4 (PAD4) plays a pivotal role in chromatin decondensation, which is one of the most crucial processes in NETs extrusion [8]. NETs can trap and kill a broad range of pathogens, including bacteria and viruses [9]. However, uncontrolled NETs formation is generally considered a double-edged sword [10]. Excessive NETs formation has been indicated to have a role in both infectious and noninfectious diseases, including but not limited to thrombosis, diabetes, vasculitis, and cancer [11, 12]. NETs are widely recognized as endogenous damage-related molecular patterns (DAMPs) that can be recognized by TLR receptors [13]. Strategies targeting NETs formation have shown therapeutic and can improve survival in animal models of sepsis [14]. Our preliminary research detected increased NETs infiltration in the intestines, and NETs disruption ameliorated intestinal injury in endotoxin mice [15]. However, the biological function and downstream signaling pathway of NETs in sepsis-induced intestinal barrier dysfunction are not yet fully understood.

The endoplasmic reticulum (ER) is the primary cellular organelle in which protein synthesis, maturation, folding, modification, and degradation take place [16]. If the optimal balances of protein folding are interrupted in the ER, a condition known as “ER stress” may occur [17]. Sustained ER stress can initiate inflammation through various mechanisms, including the production of reactive oxygen species (ROS) [18]. Quillard et al. reported that TLR2 stimulation followed by NETs participation may render smooth muscle cell-rich plaques susceptible to superficial erosion and thrombotic complications by inducing ER stress and ROS production [19]. ER stress is implicated in the progression of intestinal barrier impairments in inflammatory bowel diseases [20]. Endoplasmic reticulum stress can be regulated by TLR receptors and has a role in the process of sepsis-induced intestinal injury [21, 22].

We hypothesized that in sepsis, NETs can induce TLR receptor–ER stress–ROS signaling and activation of the inflammatory response, increasing intestinal permeability and bacterial translocation. In the current study, to our knowledge, increased local NET infiltration in the intestine in abdominal sepsis patients was firstly found, and the levels of local citrullinated histone 3 (Cit H3) were associated with the level of ER stress activation. We further determined that, in vivo, NETs depletion by PAD4 deficiency alleviated intestinal inflammation and decreased ER stress activation in a lipopolysaccharide (LPS)-induced lethal septic shock model and that, in vitro, NETs treatment induced ER stress activation and intestinal epithelial monolayer barrier disruption in a dose-dependent manner, which confirmed the detrimental effect of NETs on intestinal barrier functions. Notably, we also observed that NETs-induced intestinal barrier dysfunction was mediated by ER stress, which is regulated by Toll-like receptor 9 (TLR9).

## Results

### Increased intestinal NETs infiltration and ER stress activation in human abdominal sepsis

**Fig. 1:**
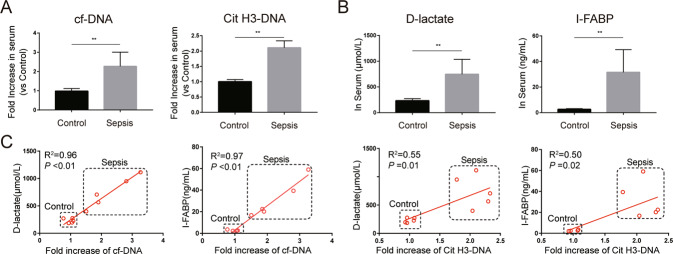
Elevated serum NETs are associated with intestinal injury in abdominal sepsis patients. **A** Circulating cf-DNA and Cit H3-DNA complexes in abdominal sepsis patients (*n* = 5) and healthy controls (*n* = 5) were analyzed by ELISA. **B** Serum I-FABP and D-lactate were analyzed by ELISA. **C** Correlation between circulating NETs biomarkers (cf-DNA and Cit H3-DNA complex) and circulating intestinal injury biomarkers (I-FABP and D-lactate). NETs neutrophil extracellular traps, cf-DNA cell-free DNA, Cit H3 citrullinated histone 3, ELISA enzyme-linked immunosorbent assay, I-FABP intestinal fatty-acid binding protein. Data are expressed as the means ± SD. *P < 0.05; **P < 0.01.

To determine NETs performance and ER stress levels in sepsis and their possible pathological impact on intestinal barrier dysfunction, we compared the relative expression in serum and relative protein levels in intestinal samples of healthy and abdominal sepsis patients. We firstly investigated whether the expression of NETs is altered in the peripheral system of these patients. Serum cell-free DNA (cf-DNA), a rough biomarker of NETs, and serum Cit H3-DNA complex, a specific biomarker of NETs, were significantly elevated in abdominal sepsis patients compared with healthy controls (Fig. 1A). In addition, the levels of serum D-lactate and intestinal fatty-acid binding protein (I-FABP), biomarkers of intestinal damage, were markedly increased in abdominal sepsis patients (Fig. 1B). Moreover, there were significant correlations between NETs markers and intestinal damage markers in serum (Fig. 1C).

**Fig. 2:**
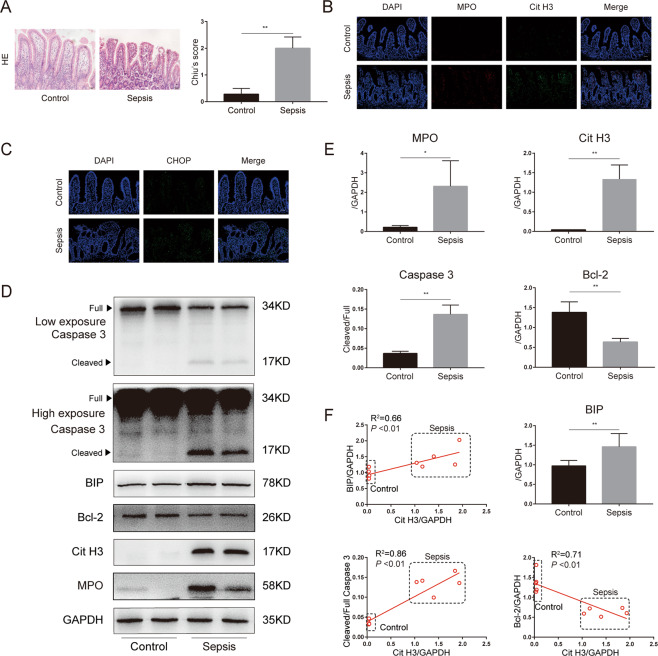
NETs infiltration was increased in the intestines of abdominal sepsis patients and correlated with ER stress activation and intestinal apoptosis. **A** Representative images of intestinal histology (HE staining) and histopathological scores (Chiu’s score) in the control group (*n* = 5) and abdominal sepsis patients (*n* = 5). **B** The expression level of NETs (MPO and Cit H3) and **C** CHOP, an ER stress biomarker, in human intestines were analyzed through immunofluorescence staining. **D, E** NETs, ER stress, and apoptotic signaling were analyzed by western blotting. ImageJ was used to detect optical density and each symbol represents an individual patient. **F** Correlation of ER stress and apoptotic signaling to the expression of NETs (Cit H3). NETs neutrophil extracellular traps, ER endoplasmic reticulum, MPO myeloperoxidase, Cit H3 citrullinated histone 3. Data are showed as the means ± SD. Scale bars = 50 μm. *P < 0.05, **P < 0.01.

Increased intestinal injuries were further confirmed by hematoxylin and eosin (HE) staining of intestines, evidenced by elevated Chiu’s scores in the abdominal sepsis group (Fig. 2A). Immunostaining analysis also demonstrated that NETs (MPO and Cit H3) infiltration was significantly elevated in abdominal sepsis patients (Fig. 2B). In addition, activation of CHOP, a biomarker of ER stress, was induced in abdominal sepsis patients compared with controls (Fig. 2C).

We then investigated the correlation of protein levels between NETs, ER stress, and intestinal apoptosis biomarkers in intestinal specimens. Compared to those in healthy controls, the protein levels of MPO, which is a biomarker of neutrophil activation and a main component of NETs, were significantly elevated in abdominal sepsis patients (Fig. 2D). In contrast to elevated levels of Cit H3 in intestinal samples from abdominal sepsis patients, only low levels of Cit H3 were detected in intestines from healthy controls. Increased cleaved caspase 3, a biomarker of apoptosis, and decreased Bcl-2, a biomarker of anti-apoptosis, were also found in human abdominal sepsis patients compared to healthy controls, and line regression analysis demonstrated a positive correlation between Cit H3 and cleaved/full caspase 3 and a negative correlation between Cit H3 and Bcl-2 (Fig. 2F).

In addition, elevated levels of BIP, a biomarker of ER stress activation, were significantly higher in abdominal sepsis patients than in healthy controls (Fig. 2D, E). Simultaneously, there was a significant correlation between Cit H3 and BIP expression (Fig. 2F). Collectively, these findings support a detrimental role of NETs in intestinal damage and a potential role of ER stress in this pathological process during human sepsis.

### PAD4 deficiency decreases systemic inflammation and organ damage in a septic shock model

**Fig. 3:**
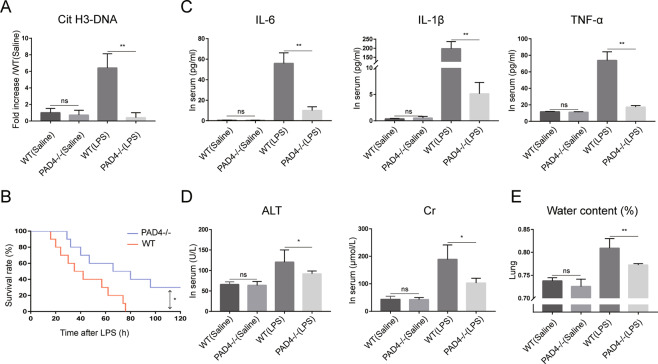
PAD4 deficiency inhibits NETs formation and decreases systemic inflammation and organ damage in septic shock. **A** Serum Cit H3-DNA complex was measured by ELISA (*n* = 6 in each group). **B** Kaplan–Meier survival analysis 120 h after LPS treatment in WT (*n* = 10) and PAD4-deficient mice (*n* = 10). **C** Serum IL-6, IL-1β, TNF-α, **D** ALT and Cr were tested by ELISA. **E** Lung water content was calculated to evaluate lung edema. PAD4 peptidylarginine deiminase 4, NETs neutrophil extracellular traps, Cit H3 citrullinated histone 3, ELISA enzyme-linked immunosorbent assay, LPS lipopolysaccharide, WT wild type, ALT alanine transaminase, Cr creatine. Data are expressed as the means ± SD. *P < 0.05, **P < 0.01.

To investigate the role of NETs in sepsis, a lethal dose of LPS was administered to induce septic shock in PAD4-deficient mice and wild-type (WT) mice. We firstly evaluated the expression of NETs in WT and PAD4-deficient mice. The Cit H3-DNA complex was markedly increased in WT mice after LPS challenge and it was abrogated in PAD4-deficient mice (Fig. 3A), which indicated that NETs formation was inhibited in PAD4-deficient mice in the LPS-induced septic shock model. Survival analysis was then carried out to evaluate the effect of NETs on post-endotoxic shock survival rate (Fig. 3B). Thirty percent of PAD4-deficient mice survived beyond 100 h and this result is in contrast to that obtained with WT mice, which all died within 80 h after LPS challenge.

We next sought to investigate whether PAD4-deficient mice are more resistant to systemic inflammation and organ damage in endotoxin shock. Serum proinflammatory cytokines, which have been shown to lead to sepsis, were detected and the concentrations of alanine transaminase (ALT) and creatinine (Cr) were used to evaluate tissue injury. The levels of serum proinflammatory cytokines (including IL-6, IL-1β, and TNF-α) significantly increased after lethal LPS challenge (Fig. 3C). In comparison, lower proinflammatory cytokines were found in PAD4-deficient mice. Moreover, WT mice also showed higher levels of serum ALT and Cr (Fig. 3D), accompanied by increased lung edema (Fig. 3E), while these organ damage markers were significantly lower in PAD4-deficient mice. Collectively, these data indicate that NETs may play a pivotal role in severe sepsis.

### PAD4 deficiency ameliorates sepsis-induced intestinal inflammation, ER stress activation, and intestinal barrier dysfunction

**Fig. 4:**
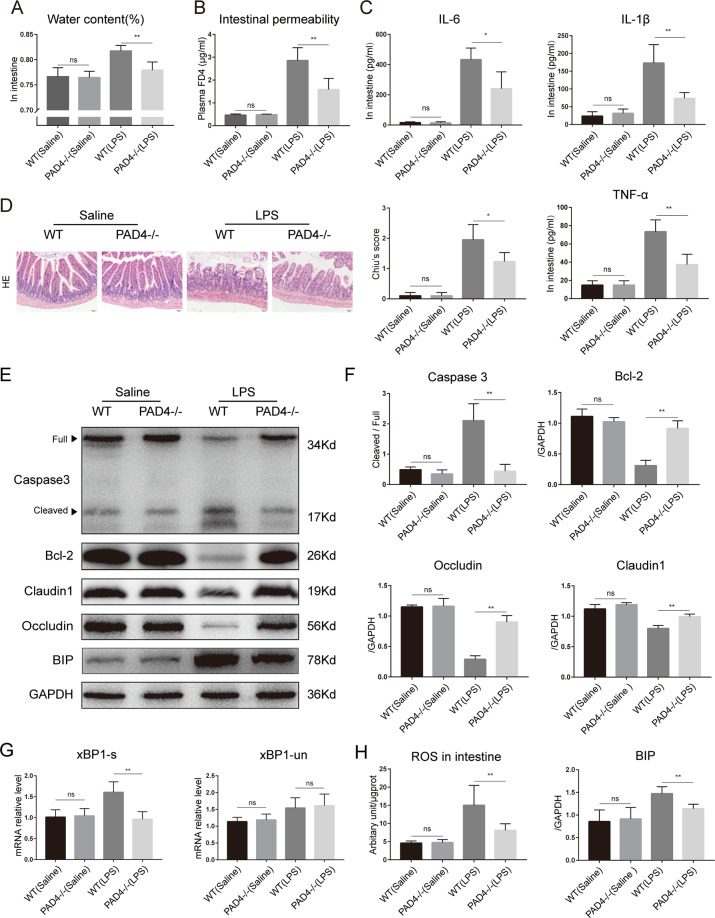
PAD4 deficiency ameliorates intestinal barrier injury and alleviates intestinal inflammation and ER stress activation in septic shock. **A** Intestinal water content was measured to analyze intestinal edema. **B** Mice (*n* = 6) in each group were fasted for 4 h and then administered FD4. Plasma FD4 was measured to evaluate in vivo permeability. **C** IL-6, IL-1β, and TNF-α in intestinal specimens were analyzed by ELISA. **D** Representative images of intestinal histology (HE staining) and histopathological scores (Chiu’s score) of the intestine after endotoxin shock. Scale bars = 50 μm. **E, F** Protein levels of tight junctions (claudin 1 and occludin), ER stress biomarker (BIP), and apoptotic signaling molecules (caspase 3 and Bcl-2) in intestinal tissue were assessed by western blotting. **G** Real-time PCR showing the variation in xBP1-s and xBP1-un mRNA expression in intestines. **H** ROS generation assessed by DCFH-DA assay. PAD4 peptidylarginine deiminase 4, ER endoplasmic reticulum, ROS reactive oxygen species. Data are expressed as the mean ± SD. *P < 0.05, **P < 0.01.

As a motor of sepsis, intestinal barrier functions were further researched. Compared to that in WT mice, decreased intestinal water content was found in PAD4-deficient mice (Fig. 4A). As expected, LPS challenge resulted in increased intestinal permeability, as evidenced by higher levels of plasma FD4 compared to the sham treatment (Fig. 4B). The level of FD4 was significantly reduced in the PAD4-deficient group. We then investigated whether NETs participate in the process of sepsis-induced intestinal inflammation and apoptosis, which contribute to increased intestinal permeability. LPS challenge induced increased local inflammatory cytokines (including IL-6, IL-1β, and TNF-α) in the intestinal mucosa of WT mice compared with sham mice (Fig. 4C). However, decreased production of these inflammatory cytokines in the intestinal mucosa was found in PAD4-deficient mice. Moreover, histological evaluation by HE staining revealed increased histological inflammatory scores in WT mice that underwent LPS challenge compared with sham mice, and there was a significant reduction in intestinal damage scores in PAD4-deficient mice compared to WT mice in LPS-induced septic shock (Fig. 4D).

Tight junction proteins play a critical role in regulating intestinal epithelial permeability and maintaining barrier functions. Therefore, the levels of tight junction proteins in the intestinal mucosa were evaluated. In this research, in LPS-induced endotoxin shock, the levels of occludin and claudin 1 were substantially increased in the PAD4-deficient group compared with the WT group (Fig. 4E). Consistently, compared to WT mice, PAD4-deficient mice exhibited decreased cleaved caspase 3 expression and increased Bcl-2 levels in the intestine. These results indicate that NETs participate in sepsis-induced intestinal apoptosis. Collectively, these findings suggested a key role of NETs in sepsis-induced intestinal barrier dysfunctions.

We then investigated whether ER stress was involved in this pathological process and compared the relative expression of ER stress biomarkers between PAD4-deficient mice and WT mice. The expression of BIP, an ER stress-related protein, in the intestine was significantly elevated in WT mice, whereas the expression in septic shock PAD4-deficient mice was lower (Fig. 4E). Simultaneously, endotoxin shock increased the mRNA expression of XBP-1s, an ER stress biomarker, in the intestine of WT mice compared with control mice (Fig. 4G). Significantly decreased mRNA levels of XBP-1s were found in PAD4-deficient mice in the LPS-induced septic shock model. Compared to control mice, WT mice showed a significant increase in ROS levels in the intestine after septic shock, and PAD4 knockout significantly reduced ROS aggregation (Fig. 4H). These findings indicated an important role of ER stress in NETs-induced intestinal injuries during sepsis.

### NETs impair the integrity of intestinal epithelial cell monolayer barriers in vitro

**Fig. 5:**
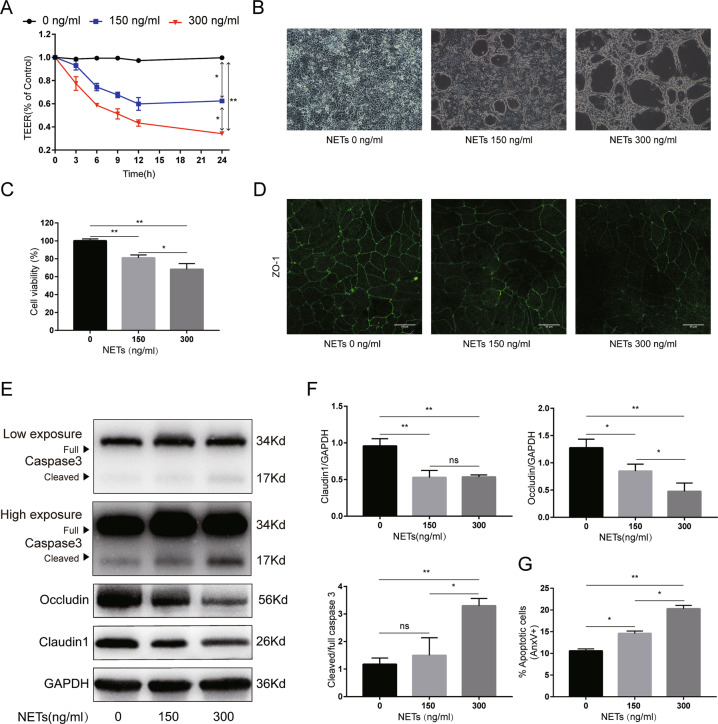
NETs dose-dependently impair Caco2 epithelial monolayer barriers in vitro. **A** Effect of NETs on the TEER values of the Caco2 epithelial barrier model. **B** The morphology of the Caco2 monolayer after NETs treatment was assessed using an ordinary optical microscope. Scale bars = 100 μm. **C** Cell viability after NETs treatment was tested by cell counting kit-8. **D** Decreased immunofluorescence localization of ZO-1 was observed in the Caco2 epithelial barrier model at 12 h after NETs treatment. Scale bars = 25 μm. **E, F** Occludin, claudin 1, and caspase3 protein expression were assessed by western blotting. The intensity was quantified using ImageJ software from three independent analyses. **G** Analysis of apoptosis (AnxV+) in the Caco2 monolayer after culture with NETs for 12 h assessed by flow cytometry. NETs, neutrophil extracellular traps. TEER transepithelial electrical resistance, AnxV annexin V. Data are expressed as the means ± SD. *P < 0.05, **P < 0.01.

To further confirm the detrimental role of NETs, we then separated NETs isolates from healthy donors and cocultured them with Caco2 monolayers or Caco2 epithelial barriers. As listed in Fig. 5A, after coculture with NETs, the transepithelial electrical resistance (TEER) values decreased in line with the NETs concentrations, indicating that NETs impaired the functions of Caco2 epithelial barriers in a dose-dependent manner. The TEER assay showed that NETs induced intestinal epithelial barrier dysfunction in a time-dependent manner within 12 h after coculture. Thus, Caco2 cells were subjected to NETs isolates for 12 h in the following experiments.

As shown in Fig. 5B, NETs disrupted Caco2 monolayers in a dose-dependent manner. Moreover, a dose-dependent cytotoxicity was also revealed, as evidenced by decreased Caco2 cell viability with increasing NETs treatment (Fig. 5C). Immunofluorescence analysis also showed that, after NETs treatment, ZO-1 localization at cellular junctions became discontinuous (Fig. 5D). Consistently, the protein levels of occludin and claudin 1 decreased and cleaved caspase 3 expression increased after NETs costimulation with Caco2 epithelial barriers (Fig. 5E). In addition, the apoptotic cell rates increased after NETs treatment (Fig. 5G). Taken together, in vitro studies confirmed the detrimental effect of NETs on intestinal barrier functions.

### ER stress regulates NETs-induced disruptions of integrity in the intestinal epithelial cell monolayer barrier

**Fig. 6:**
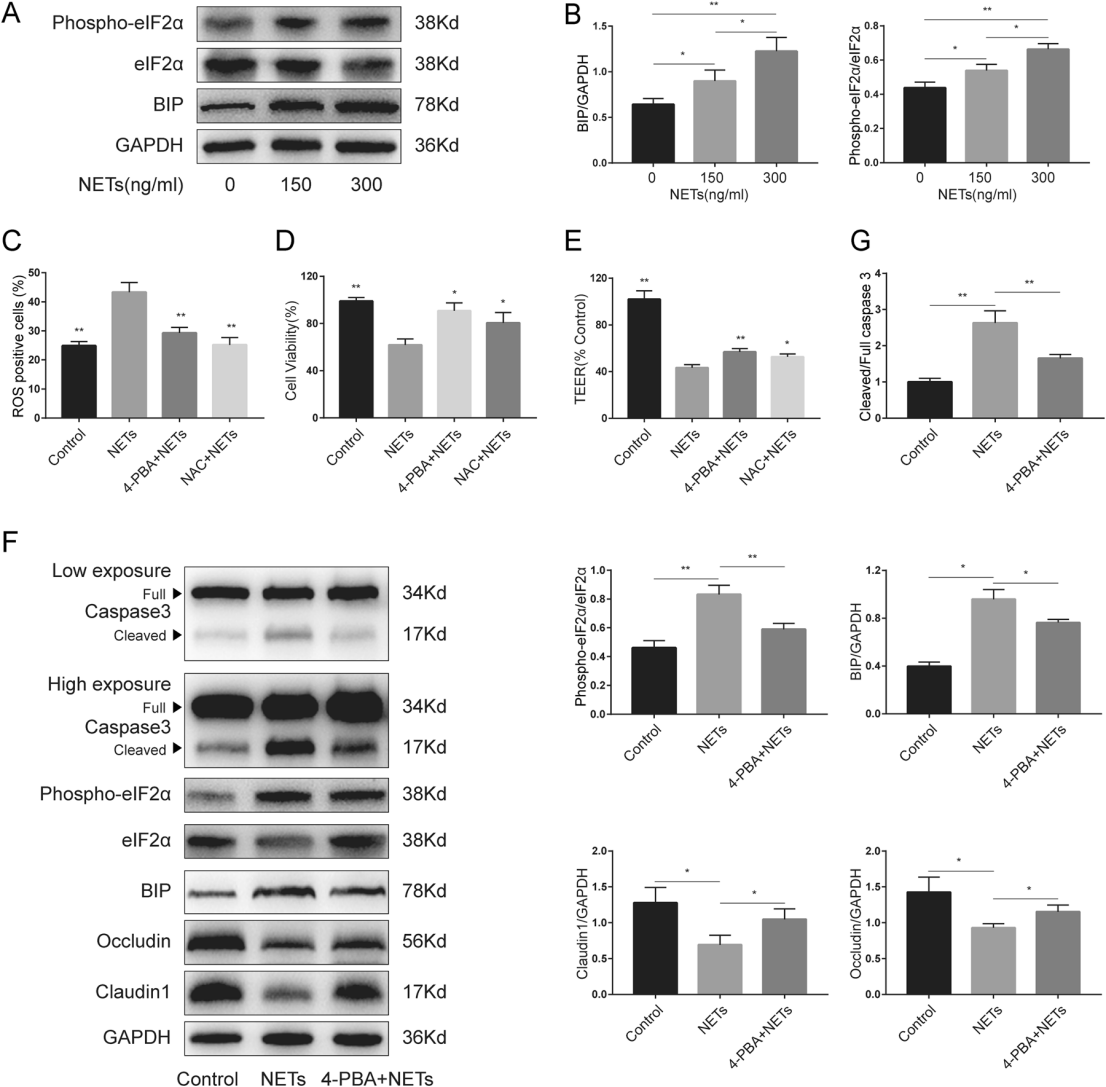
ER stress was activated by NETs, and ER stress inhibition alleviated NETs-induced damage in the intestinal epithelial cell monolayer. **A** Key molecules of ER stress were detected by western blotting in the Caco2 epithelial barrier model upon incubation with escalating doses of NETs. **B** The intensity of BIP and relative phospho-eIF2α expression were quantified using ImageJ software. **C** ROS production assessed by DCFH-DA and **D** cell viability tested by cell counting kit-8 in Caco2 monolayers after NETs (300 ng/ml) treatment with or without preincubation with 4-PBA or NAC. **E** TEER values were assessed after NETs (300 ng/ml) treatment with or without preincubation with 4-PBA or NAC. **F** The protein levels of key molecules of ER stress, caspase 3, and tight junction proteins in Caco2 epithelial barrier model after NETs (300 ng/ml) treatment with or without preincubation with 4-PBA. **G** The intensity was quantified using ImageJ software from three independent analyses. ER endoplasmic reticulum, NETs neutrophil extracellular traps, ROS reactive oxygen species, TEER transepithelial resistance. Data are expressed as the mean ± SD. *P < 0.05, **P < 0.01.

After coculturing with NETs, the expression of BIP and phospho-eIF2α increased in a dose-dependent manner in Caco2 epithelial barriers in vitro (Fig. 6A, B). In addition, in animal models, it was found that PAD4 deficiency inhibited endotoxin shock-induced ER stress activation. Moreover, PAD4 deficiency decreased sepsis-induced intestinal ROS production, and ER stress activation has been suggested to be a cause of ROS generation [23]. Therefore, we wondered whether ER stress and ROS production participate in NETs-induced epithelial injury. To further investigate the impact of ER stress and ROS in NETs-induced intestinal barrier dysfunction, 4-phenylbutyrate (4-PBA), an inhibitor of ER stress, and N-acetylcysteine (NAC), a kind of ROS scavenger, were used.

First, we found that NETs isolates treatment could induce ROS production in Caco2 cells (Fig. 6C). ER stress inhibition by 4-PBA and NAC administration inhibited NETs-induced ROS generation. In addition, both 4-PBA treatment and NAC administration significantly increased cell viability after NETs isolates treatment (Fig. 6D). Besides, TEER values were protected by 4-PBA treatment and NAC administration (Fig. 6E). Then, further research was carried out to investigate the impact of ER stress inhibition on tight junctions and epithelial apoptosis. 4-PBA administration significantly decreased ER stress-related protein levels of BIP and phospho-eIF2α (Fig. 6F). Moreover, decreased cleaved caspase 3 and increased occludin and claudin 1 levels were found after 4-PBA treatment. Collectively, the regulation of ER stress and its downstream effector ROS can alleviate NETs-induced intestinal epithelial barrier dysfunction.

### TLR9 mediates NETs-induced ER stress activation and intestinal barrier dysfunction

**Fig. 7:**
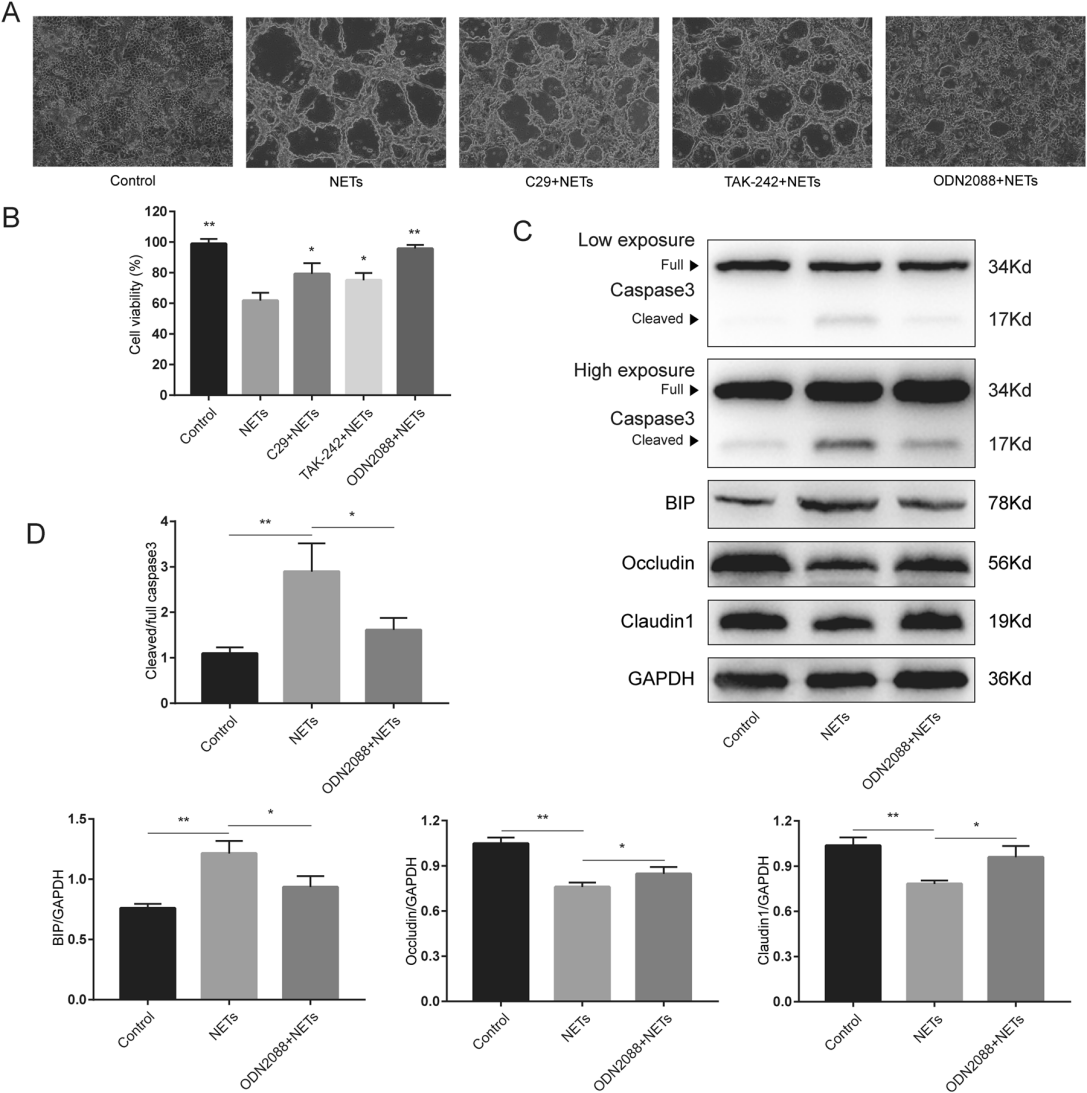
TLR9 antagonist inhibits NETs-induced ER stress activation and intestinal epithelial cell injury. **A** The morphology of the Caco2 monolayer after NETs (300 ng/ml) treatment with or without costimulation with a TLR2 antagonist (C29), TLR4 antagonist (TAK-242), and TLR9 antagonist (ODN2088). Scale bars = 100 μm. **B** Cell viability tested by the cell counting kit-8. **C** The impact of TLR9 antagonist (ODN2088) on the protein level changes in the Caco2 epithelial barrier model was assessed by western blotting. **D** The intensity was quantified using ImageJ software from three independent analyses. Data are expressed as the mean ± SD. *P < 0.05, **P < 0.01.

Histones and DNA are the major components of NETs, we next sought to determine which signaling pathway was the main intermediary between NETs and the disruption of intestinal barriers. First, Caco2 monolayers were costimulated with NETs and TLR antagonists, such as C29 (TLR2 antagonist), TAK-242 (TLR4 antagonist), or ODN2088 (TLR9 antagonist). Surprisingly, compared to administration of the TLR2 antagonist and TLR4 antagonist, administration of the TLR9 antagonist markedly alleviated NETs-induced cell morphological changes (Fig. 7A). Moreover, these three different TLR antagonists significantly increased cell viability after costimulation with NETs (Fig. 7B). Compared to TLR2 antagonist and TLR4 antagonist, TLR9 antagonist treatment more efficiently diminished NETs-induced cell injuries. Thus, we thought TLR9 may play a pivotal role in NETs-induced cell injuries, and further researches were carried out. TLR9 antagonist treatment resulted in a significant reduction in cleaved caspase 3 and an increase in occludin and claudin 1 levels (Fig. 7C). Moreover, the TLR9 antagonist efficiently reduced BIP levels after NETs isolates treatment. Collectively, these observations suggested that the TLR9–ER stress–ROS signaling pathway may contribute to NETs-induced intestinal barrier dysfunction.

## Discussion

In the present study, we firstly confirmed the increased infiltration of NETs and the correlation of NETs with intestinal injury and ER stress activation in intestinal samples from abdominal sepsis patients. Moreover, NETs directly impaired the Caco2 intestinal epithelial cell monolayer barrier in vitro and NETs inhibition by PAD4 deficiency ameliorated endotoxin shock-induced intestinal barrier dysfunction in vivo, which supported the detrimental effect of NETs in intestinal injury in sepsis. Another novel finding of the current investigation is that NETs can activate ER stress in intestinal epithelial cells and that targeting ER stress attenuates NETs-induced intestinal damage. Notably, a TLR9 antagonist reversed NETs-induced intestinal epithelial cell death and inhibited NETs-induced ER stress activation.

Excessive NETs can result in vital organ damage and serve as proinflammatory mediators in sepsis [24]. Our data revealed similar results that NETs depletion could improve the overall survival rate in LPS-induced lethal septic shock. Moreover, systemic inflammatory cytokines and vital organ damage markers were significantly reduced after NETs inhibition. Therefore, NETs regulation will be crucial in targeting organ dysfunction and the proinflammatory response in sepsis.

Circulating NETs biomarkers have been reported to be associated with prognosis in sepsis patients [25, 26]. Similar results were also found in our research, in which circulating NETs, both cf-DNA and the Cit H3-DNA complex, were significantly increased in abdominal sepsis patients. Moreover, circulating NETs had a positive correlation with biomarkers of intestinal injury. However, the contribution of NETs to sepsis-induced intestinal inflammation and gut barrier dysfunction remains to be elucidated. Our preliminary research suggested that NETs may promote intestinal injury in different animal models and observed increased formation of NETs in the intestines of critically ill surgical patients [15, 27–29]. Consistently, compared to that in healthy controls, excessive NETs infiltration was also found in intestinal samples from abdominal sepsis patients. These results indicate a potential role of NETs in intestinal epithelial injury.

Lang et al. reported that NETs degradation could ameliorate intestinal inflammation in diabetic mice [30]. In addition, Dinallo et al. showed that increased NETs were released in colonic mucosal tissues in ulcerative colitis patients and suggested a role for NETs in sustaining mucosal inflammation [31]. Li et al. demonstrated that NET regulation decreased serum inflammatory cytokines and alleviated colon tissue damage in DSS-induced colitis [32]. In an LPS-induced lethal septic shock model, we found that NETs inhibition by PAD4 deficiency decreased intestinal proinflammatory cytokines. Moreover, alleviated intestinal permeability and increased tight junction levels were also found in PAD4-deficient mice. In addition, to directly investigate the role of NETs on intestinal barrier functions, NETs were separated from human neutrophils after phorbol myristate acetate (PMA) activation and costimulate with Caco2 epithelial barriers in vitro. We found that NETs isolates treatment impaired the integrity of Caco2 epithelial cell monolayer barrier in a time- and dose-dependent manner. Collectively, these results confirmed the detrimental role of NETs in sepsis-induced intestinal barrier dysfunction.

NETs were suggested to directly induce or aggravate endothelial cell death [19, 33]. Binet et al. also reported that NETs could dose-dependently induce apoptosis in human umbilical vein endothelial cells [34]. A similar result was observed in our research: in human intestinal samples, the protein levels of Cit H3 had a significant correlation with cleaved caspase 3. With increasing NETs concentrations, the rate of apoptotic cells and the protein levels of cleaved caspase 3 increased in vitro. However, it is unclear how NETs trigger intestinal injuries. Liu et al. reported that NETs sustained the inflammatory response by inducing NLRP3 activation [13]. Meegan et al. showed that Cit H3, which is a main component of NETs, causes endothelial barrier dysfunction by reorganizing the actin cytoskeleton, and this process is not dependent on the Rho or MLCK signaling pathways [35]. In this study, increased ER stress activation was found in the intestines of abdominal sepsis patients and we also found that Cit H3 had a significant correlation with ER stress in human intestinal samples. Moreover, ER stress has been reported to be detrimental to vital organ functions in sepsis, and ER stress was suggested to promote intestinal inflammation [21, 36]. Therefore, we speculated that increased NETs infiltration activates ER stress and then promotes intestinal injury. We found that PAD4 deficiency could decrease endotoxin shock-induced ER stress activation. To further confirm the role of ER stress in NETs-induced epithelial damage, Caco2 cells were preincubated with the ER stress inhibitor, 4-PBA, before NETs isolates treatment. NETs can dose-dependently increase the level of ER stress-related proteins. ER stress inhibition alleviated NETs-induced intestinal barrier disruption and ameliorated intestinal apoptosis. Therefore, NETs exert important roles in ER stress activation, which could subsequently cause intestinal injury and intestinal barrier dysfunction in sepsis.

Excessive ROS production activates the intestinal inflammatory response and intestinal epithelial cell death [37, 38]. It is therefore of great interest to explore whether ROS mediate NETs-induced cell death. Our data showed that endotoxin shock induced intestinal oxidative stress and that PAD4 deficiency reduced ROS generation in vivo. Moreover, we demonstrated that exposure to NETs isolates triggered Caco2 cells to produce ROS. Furthermore, diminishing ROS by NAC significantly reduced NETs-induced cell death. These results suggest that ROS participate in NETs-induced intestinal injury. Excessive ROS generation can be induced by uncontrolled ER stress activation and then act downstream to trigger the inflammatory response and tissue injury [39]. In our experiment, ER stress inhibition inhibited NETs-induced ROS production. Based on these data together, we could possibly conclude that ER stress was activated by NETs and ROS was subsequently induced. Then, ROS initiated the inflammatory response and caused epithelial cell death.

Extracellular histones and DNA, the main components of NETs, are important endogenous DAMPs that induce proinflammatory signaling via TLR receptors [40]. Allam et al. demonstrated that histones were cytotoxic to renal endothelial cells and induced TLR2- and TLR4-dependent inflammatory responses [41]. Saffarzadeh et al. demonstrated the predominant role of histones in NETs-induced cell death [33]. Interestingly, Meegan et al. reported that Cit H3 could not induce cell death [35]. Moreover, DNA was previously reported as an antimicrobial component of NETs [42]. The DNA backstone contains unmethylated CpG motifs that provoke inflammatory signaling via TLR9 [43]. Moreover, Afrazil et al. demonstrated that ER stress was induced by TLR4 within intestinal stem cells, resulting in crypt apoptosis in necrotizing enterocolitis [44]. TLR2 ligands could induce endothelial ER stress in vitro and TLR9 antagonists attenuated the kainic acid-elicited ER stress response in neurons in vivo [19, 22]. In our current study, to determine which signaling pathway was mainly involved in NETs-induced disruption of intestinal barriers, antagonists of TLR2, TLR4, and TLR9 were administered. Surprisingly, although the TLR2 antagonist and the TLR4 antagonist can reduce NETs-induced cell death and morphological changes in Caco2 monolayers, a great beneficial effect was found after TLR9 antagonist costimulation with NETs isolates treatment. Moreover, the TLR9 antagonist can significantly decrease NETs-induced ER stress activation and alleviate intestinal barrier disruptions. Thus, we hypothesize that in the process of NETs-induced intestinal barrier dysfunction, compared to histones, the DNA backbone may play the predominant role in NETs directly inducing intestinal barrier dysfunction via TLR9 signaling.

**Fig. 8:**
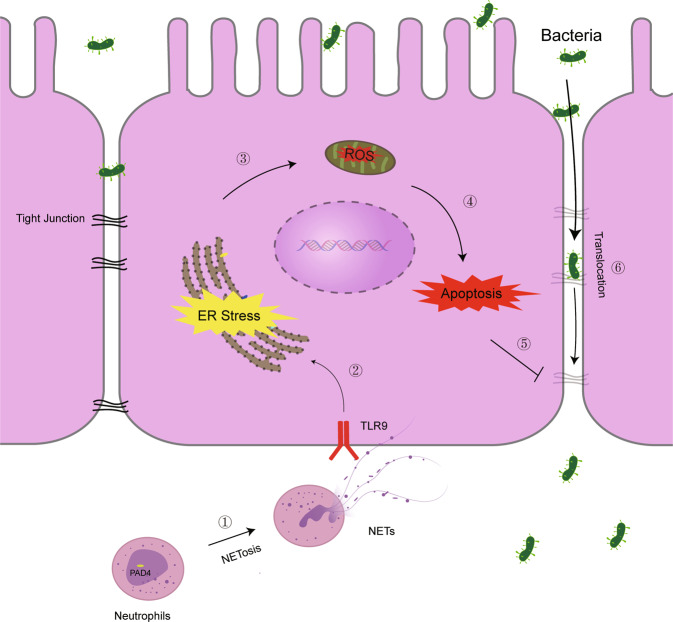
NETs induce intestinal barrier injury through the TLR9–ER stress–ROS signaling pathway. In the case of sepsis, increased neutrophils migrate and aggregate in the intestine and trigger NETosis. Excessive NETs formation can activate the TLR9–ER stress–ROS signaling pathway, which enhances intestinal epithelial cell apoptosis and the inflammatory response. Uncontrolled intestinal epithelial cell injury results in intestinal barrier disruption, leading to bacterial translocation.

In conclusion, our study revealed that NETs were involved in sepsis-induced vital organ dysfunction and intestinal injury. Increased NETs infiltration was associated with elevated intestinal inflammation and apoptosis in sepsis patients. NETs inhibition by PAD4 deficiency ameliorated sepsis-induced intestinal barrier dysfunction, ER stress activation, and ROS production. Furthermore, inhibition of ER stress and ROS alleviated NETs-induced intestinal epithelial cell death. Moreover, TLR9 antagonist significantly inhibited NETs-induced intestinal injury and ER stress activation. These findings indicated that suppression of the NETs–TLR9–ER stress–ROS signaling pathway may be protective in sepsis-induced organ injury and gut barrier dysfunction (Fig. 8).

## Methods

### Ethic statement

This study was carried out according to the Recommendations of Guidelines for Clinical Trials by the Ethics Committee of Jinling Hospital. The protocol in our study was carried out according to the principles of the Declaration of Helsinki and was approved by the Ethics Committee of Jinling Hospital (2020NZKY-011-01). Written informed consent was obtained before any study-related procedure was performed. All experiments on mice were performed in accordance with the guidelines approved by the Animal Review Committee at Jinling Hospital.

### Patients

Five adult abdominal trauma patients with confirmed intraperitoneal infection, caused by bowel perforation, leading to life-threatening organ dysfunction (Sepsis 3 definition) were enrolled in this study. Detailed clinical characteristics of abdominal sepsis patients are shown in Supplementary Table 1. Resection of the involved intestine and ostomy were performed at the surgeons’ discretion, and intestinal biopsies were obtained through stoma. Intestinal biopsies were obtained from patients, undergoing definite intestinal anastomosis of ostomy and served as controls. Informed consent was obtained from all participants.

### Animals

PAD4-deficient (C57BL/6) mice were purchased from Jackson Laboratory through the Model Animals Research Center of Nanjing University. WT (C57BL/6) mice were obtained from the Model Animals Research Center of Nanjing University. Animals were maintained under specific conditions in a temperature-controlled room. Experiments were performed with randomly chosen male mice of 8–10 weeks of age (6 in each group). To induce lethal sepsis, adult mice were injected intraperitoneally with LPS (20 mg/kg, L2880, Sigma-Aldrich). Samples were obtained at 24 h after LPS injection.

### Enzyme-linked immunosorbent assay (ELISA)

Human D-lactate (K667-100, BioVision), human I-FABP (CSB-E08024h, CUSABIO, Wuhan, China), murine TNF-α (EK282/3-96, MultiSciences Biotech, Hangzhou, China), murine IL-1β (EK201B/3-96, MultiSciences Biotech), murine IL-6 (EK206/3-96, MultiSciences Biotech), murine ALT, and Cr (Nanjing Jiancheng Bioengineering Institute) in serum or intestinal tissue were detected by using commercially available ELISA kits according to the manufacturer’s protocols.

### Quantification of NETs

To quantify NETs in mouse and human serum, cf-DNA in serum was quantified according to the manufacturer’s instructions using the Quant-iT PicoGreen dsDNA Assay kit (P11496, Invitrogen). We also developed a capture ELISA based on citrullinated histone H3 associated with DNA (Cit H3-DNA complex) according to previous research with minor modifications [45]. For the capture of antibody, 5-μg/ml anti-histone H3 (citrulline R2 + R8 + R17) antibody (ab5103, Abcam) was coated onto 96-well plates (dilution 1:500 in 50 μl) overnight at 4 °C. The plates were then blocked with 5% BSA for 2 h. After washing three times (300 μl each), 50 μl of sample was added to the wells with 80 μl incubation buffer containing a peroxidase-labeled anti-DNA mAb (Cell Death ELISA^PLUS^, 11774425001, Roche). The plate was incubated for 2 h, and shaken at 300 rpm at room temperature. After three washes (300 μl each), 100-μl peroxidase substrate (ABTS) was added. Absorbance at 405 nm was measured after 30 min of incubation at room temperature in the dark. Values for soluble NET formation are expressed as percentage increase in absorbance above control.

### Measurement of intestinal permeability and water content determination

In the intestinal permeability assay, mice were gavaged orally with FITC-dextran (FD4, MW 3000–5000, 400 mg/kg, 60842-46-8, Sigma-Aldrich) 4 h before sacrifice. Food was withdrawn from mice 4 h prior to gavage. Mice were euthanized with CO_2_ inhalation followed by cervical dislocation. Blood was collected by cardiac puncture into EDTA-K2 tubes and centrifuged at 2000 *g* for 10 min. Plasma was recovered from the supernatant and processed for measuring fluorescence intensity on a SpectraMax M5 microplate reader (Molecular Device) at 485-nm excitation/525-nm emission. A standard curve was prepared by making serial dilutions of an FD4 standard within the linear range of the curve. The concentration of FD4 in plasma was calculated using the standard curve as a reference.

Gut and lung edema were estimated by comparing tissue water content. Briefly, an ∼5-cm long ileal segment or lung tissue was harvested from each animal at 24 h after LPS administration. Tissues were dried in a 68 °C oven for 48 h. Organ water content was calculated as % H_2_O = (1 − dry weight/wet weight) × 100%.

### Histology and immunofluorescence

For histology, specimens of fresh small intestine were fixed in 10% formalin, cut into 4-μm sections, and processed on HE slides for light microscopy. A gastrointestinal pathologist expert, blinded to the experiments assessed the severity of intestinal injury using the histological score of intestine injury based on the method by Chiu et al. [46]. For immunofluorescence staining, slides were blocked with 5% bovine serum albumin in PBS at 37 °C for 1 h and the sections were incubated with 1:100 dilutions of primary antibodies, including, anti-histone H3 (citrulline R2 + R8 + R17) (ab5103, Abcam), anti-MPO (66177-1-Ig, Proteintech, Wuhan, China), anti-CHOP (15204-1-AP, Proteintech), and ZO-1 (ab96587, Abcam). Nuclei were counterstained with DAPI according to the manufacturer’s instructions.

### Isolation of human blood polymorphonuclear neutrophils and NETs isolation

Peripheral blood from healthy donors was collected in EDTA-K2 blood collection tubes, and neutrophils were isolated from whole blood using PolymorphPrep (114683, Axis-Shield) according to the manufacturer’s instructions with minor modifications. Namely, the lysis of red blood cells was performed using a hypotonic solution (NO.R1010, Solarbio, Beijing, China) according to the manuscript. Isolated neutrophils were then resuspended in RPMI medium (11875-093, Gibco) supplemented with HEPES buffer (15630-080, Gibco).

Freshly isolated neutrophils were seeded in six-well culture plates (3 × 10^6^ cells/well) and stimulated with PMA (100 nM, P1585, Sigma-Aldrich). After 4 h at 37 °C in the presence of 5% CO_2_, each well was carefully washed twice with 1-mL PBS and resuspended in DMEM. The supernatant of each well was collected and centrifuged for 5 min at 200 *g* at 4 °C to remove whole cells and debris. NETs isolates were quantified by evaluating the concentrations of DNA by the Quant-iT PicoGreen dsDNA Assay kit [47, 48].

### Cell culture and establishment of intestinal monolayer barriers

Caco2 cells were purchased from the Cell Bank of Chinese Academy of Sciences (Shanghai, China), and maintained in DMEM (10-013-CVR, Corning, China) supplemented with 10% fetal bovine serum (10099-141, Gibco), 100-U/mL streptomycin, 100-U/mL benzylpenicillin, 1% glutamine, and 1% nonessential amino acids (Gibco) in a humidified, 5% CO_2_ atmosphere at 37 °C. Cells between passages 40 and 60 were selected for experimentation to maintain relatively constant cellular phenotypes.

To create monolayer barriers, Caco2 cells were seeded in 12-well transwell plates, on polyester membrane filters (pore size 0.4 μm, surface area 1.12 cm^2^, 12-mm diameter; 3401, Corning Costar Corporation) at a density of 1 × 10^5^ cells/cm^2^ with 0.5-mL culture medium on the apical side and 1.5-mL culture medium on the basolateral side. The media were replaced every 2 days for the first 7 days and then daily thereafter. The cells grew for 21 days to build a confluent monolayer.

The integrity of the Caco2 monolayers was evaluated by the TEER. The TEER was measured by using an epithelial voltohmmeter (EVOM2, World Precision Instruments, USA). The TEER value was calculated using the following equation: TEER value = [Ω cell monolayer – Ω filter (cell-free)] × filter area. The monolayer with a TEER value >400 Ω * cm^2^ was considered qualified for the following tests. The TEER values were normalized to the initial values, and expressed as percentages of the initial resistance values.

### Measurement of ROS production

The fluorescent probe 2′,7′-dichlorofluorescein diacetate (DCFH-DA, E004-1-1, Nanjing Jiancheng Bioengineering Institute) was used to detect the ROS level according to the manufacturer’s instructions with minor revisions.

For detection of intestinal ROS expression, fresh ileum tissue (0.1 g of each sample) was cut into 1-mm pieces, homogenized in PBS, and centrifuged twice for 5 min each (1500 rpm), and the supernatant was subsequently discarded. The solution was digested using trypsin at room temperature for 5 min, and digestion was terminated by the addition of a small amount of serum. Subsequently, the solution was filtered through a 100-μm filter, and 10-mL PBS was used to rinse the tissue. The filtrate was collected and centrifuged twice (5 min, 4 °C, 1500 rpm), and the residual pellet was collected and resuspended in 10-µM DCFH-DA in serum-free DMEM. An hour later, the fluorescence was measured at excitation wavelengths of 485 nm and emission wavelengths of 530 nm with a SpectraMax M5 microplate reader (Molecular Device).

For detection of intracellular ROS, Caco2 cells (5 × 10^5^ cells per well) were plated on six-well plates for 24 h. Caco2 cells were preincubated with different inhibitors for 1.5 h followed by treatment with NETs isolation for 12 h. Subsequently, intracellular ROS were detected by flow cytometry.

### Analysis of apoptosis and cell viability

The FITC Annexin-V Apoptosis Detection Kit (556547, BD Biosciences) was used to detect apoptosis in Caco2 cells following the manufacturer’s instructions. Briefly, after specific treatment with or without NETs isolation, cells were collected and washed three times with cold PBS, and then resuspended in 100-μl binding buffer, to which 5-μl Annexin V and 5-μl PI were added. After incubation in dark for 15 min at room temperature, the cells were diluted with 200-μl binding buffer. Finally, the apoptotic cells were analyzed on a FACS flow cytometer (BD Biosciences).

Cell viability was determined using a commercially available Cell Counting Kit (CCK-8) (C0038, Beyotime, Shanghai, China) according to the manufacturer’s instructions. Briefly, Caco2 cells were seeded in a 96-well plate at a density of 5 × 10^4^/well and maintained in DMEM for 24 h at 37 °C. Caco2 cells were preincubated with or without various inhibitors for 1.5 h, such as ER stress inhibitor (4-PBA, 5 µM, HY-A0281, MedChem Express), TLR2 inhibitor (C29, 50 µM, HY-100461, MedChem Express), TLR4 inhibitor (TAK-242, 1 µM, HY-11109, MedChem Express), TLR9 inhibitor (ODN2088, 5 µM, tlrl-2088, InvivoGen), or NAC (10 mM, A7250, Sigma-Aldrich). Subsequently, the cells were exposed to NETs isolates at different concentrations. Following incubation for 24 h at 37 °C, 10 µl/well CCK-8 solution was added to the medium and cultured for 3 h at 37 °C. Subsequently, the absorbance of each well at 450 nm was determined using SpectraMax M5 microplate reader (Molecular Device). Each sample was analyzed in triplicate, and the mean absorbance was calculated as the final result.

### Western blot analysis

Total proteins in the intestinal tissues of mice with LPS-induced sepsis or cells were extracted using lysis buffer (SD001, Invent Biotechnologies). Proteins were separated by SDS-PAGE and transferred to PVDF membranes. The membranes were blocked with 5% nonfat milk for 2 h and then incubated overnight at 4 °C with specific primary antibodies, including anti-histone H3 (citrulline R2 + R8 + R17) (ab5103, Abcam), MPO (66177-1-Ig, Proteintech), Bcl-2 (26593-1-AP, Proteintech), occludin (ab216327, Abcam), claudin 1 (ab15098, Abcam), caspase 3 (9662S, Cell Signaling Technology), BIP (11587-1-AP, Proteintech), phospho-eIF2α (3398T, Cell Signaling Technology), and eIF2α (5324T, Cell Signaling Technology). The next day, membranes were incubated with HRP-conjugated secondary antibody, and protein bands were visualized by using ECL. Protein quantification was measured in optical density units using Image J or Image Lab software (Bio-Rad) and was normalized to the corresponding sample expression of GAPDH.

### Quantitative real-time PCR analysis

Total RNA was extracted from intestinal tissue using TRIzol reagent (15596026, Invitrogen) according to the manufacturer’s instructions, and its purity and concentration were determined by a Nanodrop instrument (Agilent Technologies). Single-strand cDNA was generated by reverse transcription using HiScript II QRT SuperMix for qPCR (R223-01, Vazyme, Nanjing, China) according to the manufacturer’s instructions, and relatively quantitative real-time PCR was performed using ChamQ SYBR Color qPCR Master Mix (Q431-02, Vazyme) and a Fast 7500 real-time PCR system (Applied Biosystems), all according to the manufacturers’ manuals. Fold changes in the expression levels of targets were calculated by the 2^−ΔΔCT^ method using GAPDH as an endogenous reference. The PCR was run in duplicate for each sample. Primers used in qRT-PCR were synthesized by TSINGKE Biological Technology (Nanjing, China). A list of the primers used in these studies is provided in Supplementary Table 2.

### Statistical analysis

The related results were shown as the mean ± standard deviation or median with interquartile range. At least three independent experiments were conducted to confirm the results and differences between groups were tested by the Student’s *t*-test or one-way analysis of variance. Statistical analyses were conducted using GraphPad Prism software 7.0. *P* < 0.05 was considered statistically significant (*P < 0.05; **P < 0.01).

## Supplementary information

Supplementary Table 1

Supplementary Table 2

## Data Availability

The datasets used and/or analyzed during the current study are available from the corresponding author on reasonable request.
