# Supplementary material for: Neutrophil extracellular traps impair intestinal barrier functions in sepsis by regulating TLR9-mediated endoplasmic reticulum stress pathway
Source: Cell Death Dis. 2021 Jun 11;12(6):606. doi: 10.1038/s41419-021-03896-1 (PMC8195983; doi:10.1038/s41419-021-03896-1)
Supplement: Supplementary file 1 — Supplementary Table 1 [file 41419_2021_3896_MOESM1_ESM.docx]

| Supplementary Table 1: Clinical characteristics of abdominal sepsis patients and healthy controls | | |  |
| --- | --- | --- | --- |
|  | Control group (n=5) | Abodminal sepsis group (n=5) |  |
|  |  |  |  |
| Age, mean±SD | 40.6 ± 6.9 | 39.0 ± 8.3 |  |
| Gender, male, n (%) | 4(80) | 4(80) |  |
| SOFA score, mean±SD | N/A | 5.8 ± 1.5 |  |
| APACHE II score, mean±SD | N/A | 12.8 ± 3.1 |  |
| Mechanical ventilation, n (%) | N/A | 3(60) |  |
| White cell counts, *10^9^/L, mean±SD, | 6.8 ± 1.5 | 19.4 ± 4.7 |  |
| Neutrophil counts, *10^9^/L, mean±SD | 3.5 ± 0.8 | 16.7 ± 5.1 |  |
| C-reactive protein, mean±SD, mg/L | 1.1 ± 0.8 | 133.4 ± 47.3 |  |
| Time of ICU stay, mean±SD, day | N/A | 16.2 ± 2.3 |  |
| Time of hospital stay, mean±SD, day | 20.0 ± 5.8 | 37.0 ± 11.1 |  |
