# Supplementary material for: Neutrophil extracellular traps impair intestinal barrier functions in sepsis by regulating TLR9-mediated endoplasmic reticulum stress pathway
Source: Cell Death Dis. 2021 Jun 11;12(6):606. doi: 10.1038/s41419-021-03896-1 (PMC8195983; doi:10.1038/s41419-021-03896-1)
Supplement: Supplementary file 2 — Supplementary Table 2 [file 41419_2021_3896_MOESM2_ESM.docx]

| Supplementary Table 2: Primers of the genes used in the study. | | |
| --- | --- | --- |
| Genes | Forward | Reverse |
| GAPDH | AGGAGCGAGACCCCACTAACA | AGGGGGGCTAAGCAGTTGGT |
| x-BP1-s | TGAGTCCGCAGCAGGTGC | CAACTTGTCCAGAATGCCCAAAAGG |
| x-BP1-un | AAGAACACGCTTGGGAATGGACACGC | ACCTGCTGCAGAGGTGCACATAGTC |
